# Bisphenol A triggers apoptosis in mouse pre-antral follicle granulosa cells via oxidative stress

**DOI:** 10.1186/s13048-023-01322-y

**Published:** 2024-01-16

**Authors:** Chen Wang, Chaofan He, Shumin Xu, Yuanyuan Gao, Kaixian Wang, Meng Liang, Ke Hu

**Affiliations:** School of Life Science, Bengbu Medical University, Bengbu, 233030 China

**Keywords:** Bisphenol A, mpGCs, Reactive oxygen species, Oxidative stress, Apoptosis

## Abstract

**Background:**

Bisphenol A (BPA), an endocrine disrupting chemical with weak estrogenic and anti-androgenic activity, is widely present in various environmental media and organisms. It has certain reproductive toxicity and can cause a variety of female reproductive system diseases. Although BPA-stimulated apoptosis of granulosa cells has been widely elaborated, the effect of BPA on mouse pre-antral follicle granulosa cells (mpGCs) has not been well elucidated.

**Results:**

In this study, the results of live-dead cell staining showed that high concentrations of BPA severely impaired mpGCs growth viability and affected the cell cycle transition of mpGCs. We confirmed that BPA promotes the production of reactive oxygen species (ROS) and facilitates oxidative stress in mpGCs. In addition, immunofluorescence, transmission electron microscopy, and flow cytometry experiments demonstrated that BPA treatment for mpGCs resulted in apoptotic features, such as rounding, cytoplasmic crinkling, and mitochondrial damage. This was accompanied by a large production of ROS and apoptosis-inducing factor (AIF) translocation from the mitochondria to the nucleus. RNA-seq data showed that several apoptosis-related pathways were enriched in the high concentration BPA-treated group compared with the normal group, such as the p53 pathway, MAPK pathway, etc.

**Conclusions:**

These results suggest that cells undergo oxidative stress effects and apoptosis after BPA treatment for mpGCs, which affects normal follicle development. The potential mechanism of BPA-induced female reproductive toxicity was elucidated, while providing a research basis for the prevention and treatment of female reproductive diseases.

**Supplementary Information:**

The online version contains supplementary material available at 10.1186/s13048-023-01322-y.

## Introduction

In recent years, a category of environmental toxins that affect the endocrine system of the body and adversely affect its function have attracted people’s attention, and researchers have defined them as environmental endocrine disrupting chemical (EDC) [1]. The biological structure of EDC is similar to that of endocrine steroid hormones, so they can mimic steroid hormones and bind directly to hormone receptors to affect the synthesis, secretion, transport, and metabolism of endocrine hormones, thus interfering with the normal function of the human endocrine syste [2, 3]. Bisphenol A (BPA) is an important chemical raw material that is extensively used in industrial production and daily life as an EDC and has caused some harm to the ecological environment due to its widespread use and the characteristics of difficult degradation and easy enrichment [4]. At the same time, the chemical structure of BPA has two phenolic hydroxyl functional groups, similar to estradiol, and when it enters the body, it works by binding to estradiol receptors, interfering with hormone metabolism in the body and affecting the reproduction and development of the body [5]. Typically, humans are exposed to BPA through enrichment in the food chain; in general, BPA in the environment also enters the body through the skin or respiratory tract [6].

BPA exposure has become a growing social problem, and although studies have shown that prenatal, perinatal, and postnatal exposure can impair processes related to ovarian development, induce abnormal ovarian morphology, and impair ovarian function in female adult animals and offspring, early BPA exposure can even show across generations [7]. Follicles play an important role in female reproductive development [8]. Therefore, mpGCs in follicles attracted our attention. We tried to reduce the damage of EDC to human body by exploring the relationship between mpGCs and BPA. Population exposure monitoring in countries around the world has shown that BPA can be detected in follicular fluid, blood, urine, placenta, breast milk, and other body fluids or tissues, and the detection rate in urine is as high as 95% [9–11]. It is now widely believed that women have significantly higher levels of BPA in their urine than men, which may be due to women's greater exposure to related plastic products such as cosmetics [12]. Furthermore, BPA has been detected not only in serum and urine samples from pregnant women, but also in amniotic fluid, cord blood, placental tissue, and urine samples from newborn infants [13–15]. In addition, the level of BPA in maternal amniotic fluid is five times higher than that in maternal serum during pregnancy [13, 16]. This suggests that BPA can accumulate in the embryo through the placental barrier and may affect fetal development due to maternal exposure to varying levels of BPA [17, 18]. In particular, it has been shown that BPA shows a high affinity for the endoplasmic reticulum and can mimic the estradiol-releasing action process, thereby stimulating estradiol -induced function [19, 20]. Because BPA has the properties of stimulating estradiol receptor, it is speculated that it is associated with a variety of female reproductive system diseases, and estradiol receptor-dependent gene expression is closely related to the normal function of female reproductive system [21, 22]. A dose-response relationship between BPA exposure and altered maturation of human oocytes in vitro has been reported [23]. mpGCs are the main components of the follicle and promote oocyte maturation by secreting estradiol and providing nutrients [24]. So mpGCs play an important role between follicles, estradiol and female reproductive function. Once mpGCs are damaged by the outside world, follicle development will be hindered, and estradiol cannot play its normal role, leading to female fertility decline and infertility. BPA can inhibit the transition of mouse mpGCs from G2 stage to M stage and induce cell apoptosis. Therefore, mammalian mpGCs play an important role in regulating hormones and nutrients during the early development of follicles [25]. On this basis, this study has certain clinical significance. Oxidative stress is the most common reaction in cells, which can cause different degrees of cell death. However, the regulation of BPA on mpGCs oxidative stress in mice has not been reported.

Studies have found that BPA can cause oxidative stress response, interfere with the synthesis of ovarian steroid hormones, inhibit oocyte maturation, and lead to follicle apoptosis [26]. BPA enhanced the expression of cycle-related proteins Cdk4, Ccnel, and Trp53 in caved follicles, decreased the expression of Ccnd2, caused cell cycle disorder, significantly increased the expression of apoptosis-related proteins Bax and Bcl-2, and promoted the atresia of the antral follicles. In vitro cultures of mouse oocytes show spindle aberrations and abnormal chromosome integration after treatment with high concentrations of BPA. Also, BPA intake in mice induced DNA methylation and DNA damage [27]. Studies have demonstrated that BPA induces ROS production and Ca^2+^ accumulation in KGN cell line, the KNG cell line is derived from human ovarian granulosa cells [11]. However, there are almost no relevant reports in primary cultured mpGCs, the objective of this study is to explore the interaction between BPA and mpGCs, the mechanism of mpGCs apoptosis after BPA exposure in mpGCs, and clarify the potential mechanism of female reproductive disorders induced by EDC, so as to provide an entry point for the prevention and treatment of infertility.

## Materials and methods

### Reagents

The cell cycle detection kit (Beyotime Biotechnology, Shanghai, China), TUNEL kit (Beyotime Biotechnology), reactive oxygen species detection reagent (Beyotime Biotechnology), and BPA (Solarbio Technology, Beijing, China) were purchased from the appropriate biological companies.

### Animals and cell cultures

ICR mice were provided by the Skbex Biotechnology Co., LTD. Female mice at 10–12 days were selected, and mpGCs were isolated from the ovaries according to the previous experimental method [28, 29]. The specific separation procedures were as follows: ICR female mice were killed by cervical dislocation 10-12 days after birth, the abdominal cavity was opened, the ovarian tissue was removed, and the follicles in the diameter range of 120-150 μM were separated with a few fine needles (Sigma-Aldrich, Louis, MO). It is then digested in a liquid containing type IV collagenase (Sigma-Aldrich)and trypsin-EDTA (Life Technologies, Carlsbad, CA). The isolated cells were then subjected to subsequent cell culture experiments. The isolated pellet cells were cultured in 5% CO_2_ and a 37 °C incubator with a DMEM/F12 medium with 10% FBS and 1% double antibiotics. After 12 h, the suspended cells were removed by replacing the medium with fresh medium. Then, the medium was changed every two days and mpGCs were collected for study immediately after the cells were spread over the culture dish. The cell culture process is the same and the operation is repeated three times. This study was authorized by the Experimental Animal Ethics Committee of Bengbu Medical University.

### Cell cycle assay

mpGCs was starved with 6-well plates for 24 h and treated with different concentrations of BPA for 12 h. The experiment was set up in 0 μM BPA control group and 10, 50, 100, 150, 200 μM BPA treatment group. 5 mg bisphenol a was prepared in 500 μl anhydrous ethanol to prepare a specific concentration of the stock solution. According to the quantity conservation relationship of chemical substances, the final required concentration was obtained, and the stock solution with specific concentration of BPA per well was respectively added into the six-well plate to be 0 μl, 0.228 μl, 1.14 μl, 2.28 μl, 3.42 μl, 4.56 μl and 1 ml culture solution, for subsequent cell culture and experimental operations. 0.5 ml of trypsin was added to each well for digestion of adherent cells and 3 min later, cell culture solution was added to terminate the digestion and collect the cells in 1.5 ml centrifuge tubes. Pre-chilled PBS was used to wash and collect the cells by centrifugation. The cells were resuspended in pre-chilled 70% ethanol, gently inverted and mixed, fixed at 4 °C for 3 h, and collected by centrifugation. The required volume of staining solution was prepared according to the instructions of the cell cycle assay kit. 0.5 ml of staining solution was added to the samples, stained for 30 min at 37 °C, and protected from light. The cell cycle was detected using flow cytometry.

### Live-dead cell staining

We verified the cell viability after BPA treatment by live-dead cell staining. In six-well plates, mpGCs (after 12 h of treatment) were washed twice with PBS. One ml of Calcein-AM staining working solution was added to each well and incubated at 37 °C for 30 min in a light-proof environment. The serum-free medium was replaced and the solution was incubated for another 30 min away from light. Replace with 1 ml of Propidium iodide (PI) working solution and incubate for 10 min. after incubation, observe by fluorescence microscope and record the results.

### Terminal deoxynucleotidyl transferase-mediated dUTP-biotin nick end labeling (TUNEL) assay and immunofluorescence

The mpGCs were passage into 24-well plates for 24 h and given different concentrations of BPA treatment for 12 h. The old culture medium was aspirated and the well plates were washed twice with PBS. Cells were then fixed with 0.5 ml of 4% paraformaldehyde for 20 min and then washed once with PBS. Then add 0.5 ml of 0.3% Triton X-100 permeabilized at room temperature for 5 min, wash once with PBS, then configure TUNEL assay solution strictly according to the instructions, add 0.1 ml of assay solution to each well, mix thoroughly, incubate for 1 h at 37 °C protected from light, wash the well plate twice with PBS after staining, add 4,6-diamidino-2-phenylindole (DPAI) staining for 1 min to intercross cell nuclei, PBS washed 2 times, 0.5 ml of PBS was added to each well, and fluorescence microscopy was taken to observe and record apoptotic cells.

The pre-treatment of the immunofluorescence experiment was consistent with the TUNEL experiment. After permeabilization, the plates were sealed at room temperature for 0.5 h. Subsequently, the plates were incubated overnight at 4° with AIF antibody diluent (Abclonal Technology, Wuhan, China). After incubation with primary antibody, the plates were washed twice with PBS and incubated with fluorescent secondary antibody for 1 h. The plates were washed twice with PBS, the nuclei were labeled with DPAI staining solution for 1 min and washed twice with PBS. 0.5 ml of PBS was added to each well, and fluorescence microscopy was taken for observation.

### Intracellular ROS detection experiment

mpGCs were passed to 6-well plates for 24 h and given gradient concentrations of BPA for 12 h. The cell culture fluid was removed, and the cells were washed twice with PBS. One ml of 1000 × DCFH-DA working solution was added. The cells were stained at 37 °C for 20 min, the serum-free medium was washed three times, and the ROS signal was detected by fluorescence microscopy photo observation or flow cytometry.

### Western blot

Total proteins from mpGCs treated with different concentrations of BPA were extracted with RIPA lysate containing 1% cocktail, and then Western blot assay was performed. The specific operation process is as follows: First, according to the protein concentration detection, it can be seen that its concentration is 20-25 μg/ml, and the protein amount of the control group is quantified, so the sample amount of the experimental group is adjusted to ensure that the total amount of protein added to each hole in the later stage is the same. A proportional gel was prepared using the SDS-PAGE gel kit (Beyotime), followed by a 120 V electrophoresis with the calculated loading amount, followed by a 250 mA electrofolk membrane, which was transferred to the nitrocellulose membrane (Amersham Biosciences, Freiburg, Germany). At the end of the transfer, they were enclosed in skim milk powder and incubated with γ-H2AX and β-actin antibodies (Abclonal). The dilution ratio of γ-H2AX antibody is 1:250, and the dilution ratio of β-Actin antibody is 1:1000. Finally, the results of γ-H2AX protein development were compared with the internal reference results for standard quantification.

### High-throughput sequencing

Based on the above experimental results, we finally selected 200 μM treatment for transcriptomic analysis 12 h after treatment, because of various experimental results such as ROS, apoptosis and electron microscopy in this treatment group. It's more about what we're trying to do. RNA samples were extracted from the control and the high-dose BPA group. The process of RNA extraction is as follows: 1 ml Trizol (Invitrogen, California, USA) reagent is added for full cracking, and the cracked sample or homogenate is placed at room temperature for 5-10 min, so that the nucleoprotein and nucleic acid are completely separated. Add 0.2 ml chloroform (Macklin, Shanghai, China), shake violently for 15 s, leave at room temperature for 3 min, then centrifuge at 12000 rpm at 4 °C for 10 min; Absorb the upper transparent water phase and transfer it to a clean centrifuge tube, add isopropyl alcohol (Macklin) in equal volume mix well, leave at room temperature for 20 min, centrifuge at 12000 rpm at 4 °C for 10 min, discard the supernatant; Add 1 ml 75% ethanol (Macklin) for washing and precipitation, centrifuge at 12000 rpm at 4 °C for 3 min, discard the supernatant, and dry at room temperature for about 5 min. Add 30–50 μl RNase-free ddH2O (Beyotime), fully dissolve RNA, and store the RNA solution at -80 °C after concentration and instrument detection (Accurate, Changsha, China). After the quality assessment of the original sequencing data, relatively accurate and effective data can be obtained. After RNAseq sequencing and gene structure analysis, we can select transcription regions to evaluate gene expression levels by using a consistent gene model. For expression difference analysis, DESeq should be used to visualize the expression difference analysis results, map the differential genes to the STRING protein interaction network database for protein interaction network construction, and draw Wayne map and heat map based on the difference analysis results, and perform cluster analysis. Finally, functional enrichment analysis of genes was obtained. The differentially expressed genes were screened for Gene Ontology (GO) and Kyoto Encyclopedia of Genes and Genomes (KEGG) enrichment (screening conditions: *P* ≤ 0. 05 and |Log2Fold Change|≥ 1) by the Illumina platform of Sangon Biotech (Shanghai).

### Statistical analysis

Data were statistically processed using SPSS 24.0 software and expressed as mean ± standard deviation, and one-way ANOVA was used to analyze the differences between groups. All data conform to normal distribution, with *P* < 0.05 indicating that the differences were statistically significant.

## Results

### Verification of cell viability after treatment with gradient concentrations of BPA

**Fig. 1:**
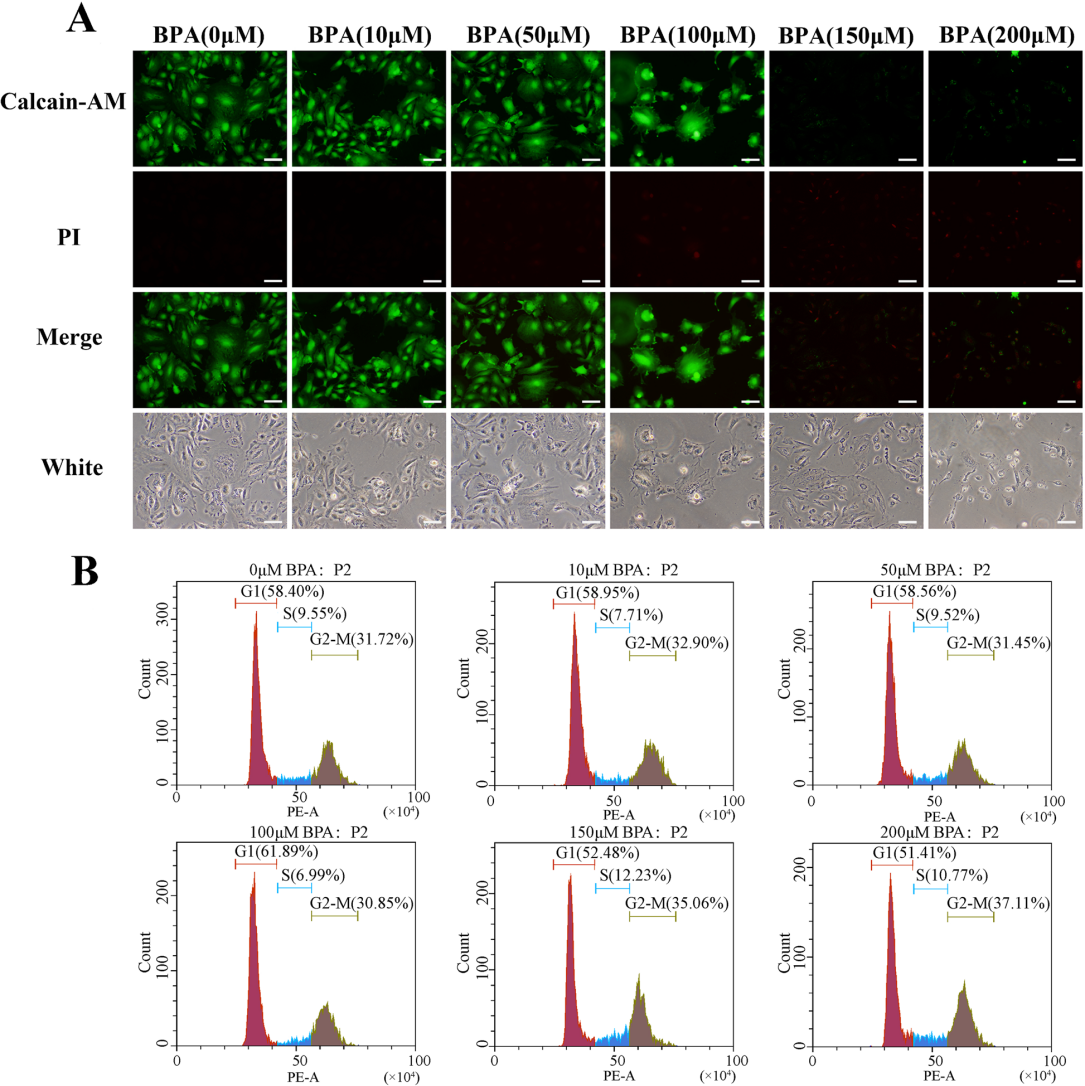
Verification of cell viability after treatment with gradient concentrations of Bisphenol A (BPA). After treating pre-antral follicle granulosa cells (mpGCs) with different gradient concentrations of BPA at 0, 10, 50, 100, 150, and 200 μM for 12 h, cells were collected for different experiments. **A** Live-dead cell staining showed a dramatic decline in cell viability in the group treated with high concentrations. **B** The cell cycle results showed that the proportion of cells in the G1 phase was greatly reduced

**Fig. 2:**
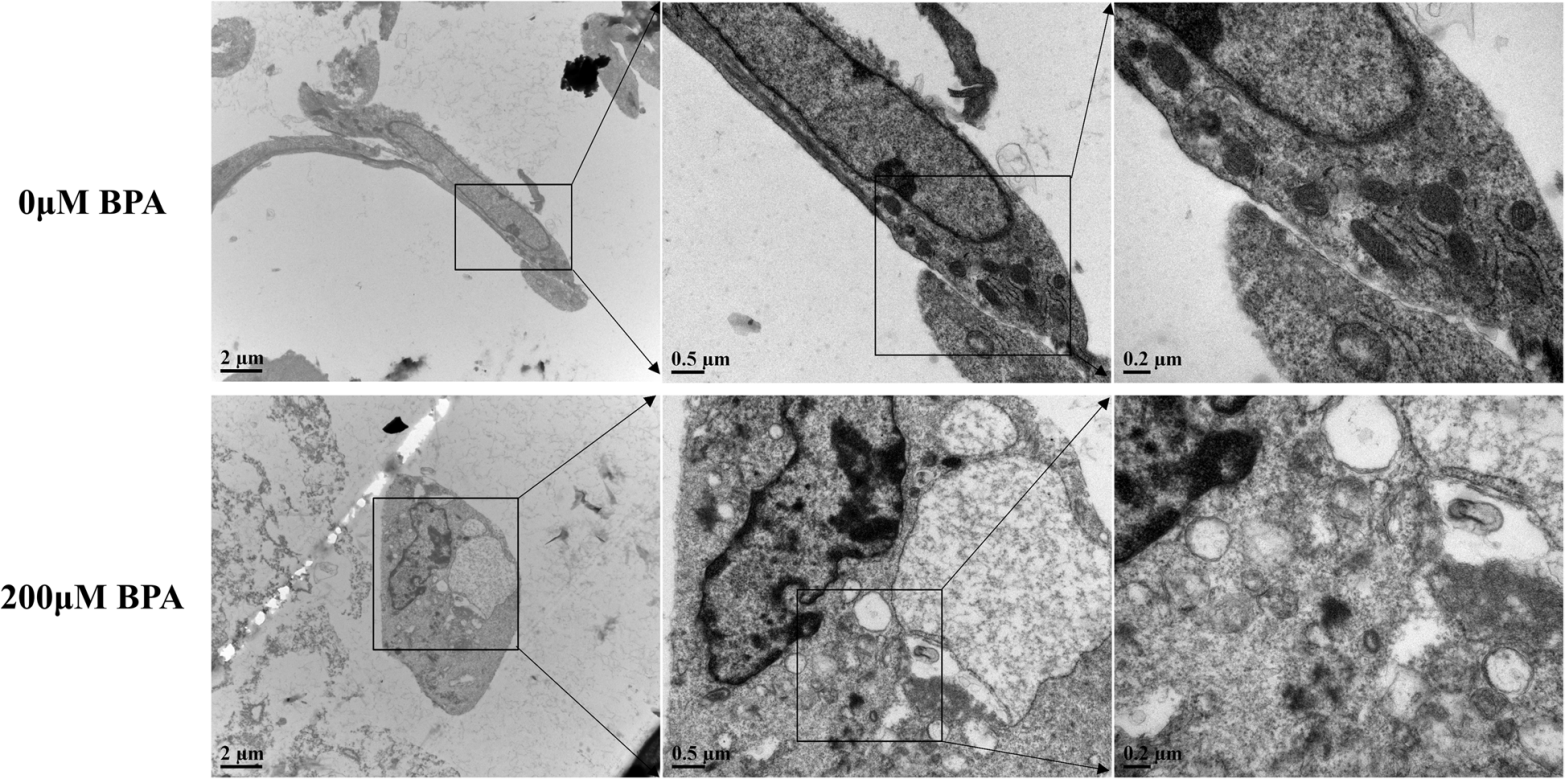
Microstructure of mpGCs treated with high concentrations of BPA by projection electron microscopy. Cells in the treated group (12 h treatment of 200 μM BPA) showed mitochondrial damage and vacuole production compared with the control group

A large number of data show that the accumulation of BPA can cause serious damage to the body, and we chose 12 h as the experimental treatment time in this study to find out whether BPA has caused some changes in molecular mechanisms in the body within a short time, because biochemical reactions are a process of effect accumulation. If we can block BPA in a short period of time, it will have certain significance for subsequent clinical prevention and treatment, and we will continue to extend the time point for further research. Our previous study showed that low concentrations of BPA promoted the proliferation of mpGCs, whereas high concentrations of BPA inhibited the proliferation of mpGCs, and the growth curves of mpGCs treated with gradient concentrations of BPA showed an inverted "U" shape [30]. In this study, we further observed the effect of BPA on the cell viability of mpGCs by live-dead cell staining. We found that the cell viability of mpGCs decreased with increasing concentration after BPA exposure, and PI-positive cells had a significant increase (Fig. 1A). In the experiment of cell cycle, the proportion of G1 phase in the control group was 58.40%, and the proportion of G1 phase in the experimental group at 150 μM and 200 μM was 52.48% and 51.40%, respectively. Statistical analysis showed that there was a significant difference compared with the control group. The same is true for the S and G2-M phases. (Figure 1B). Statistical analysis of relevant data also accords with the above conclusion (Supplemental Fig. 1). In terms of the choice of concentration, considering that this paper explores the damage related to BPA exposure, we tend to choose a slightly higher concentration group to better simulate the effect caused by absorption in vivo. In addition, we finally determined the concentration groups by referring to previous studies [31]. Transmission electron microscopy results showed that cells in the 200 μM BPA-treated group had rounded morphology, incomplete nuclear membranes, and mitochondrial damage compared to the normal group (Fig. 2). It is suggested that after BPA exposure, mpGCs may undergo intense intracellular activity leading to mitochondrial damage, which in turn promotes apoptosis. Meanwhile, we used a live cell workstation to continuously observe the cell status of mpGCs after BPA exposure; From the 10h, PI-positive cells were already present and the cells gradually crumpled, and finally after 30 h of continuous observation, most of the cells underwent apoptosis (Supplemental Video 1).

### BPA drives mpGCs to produce large amounts of ROS and induces apoptosis

**Fig. 3:**
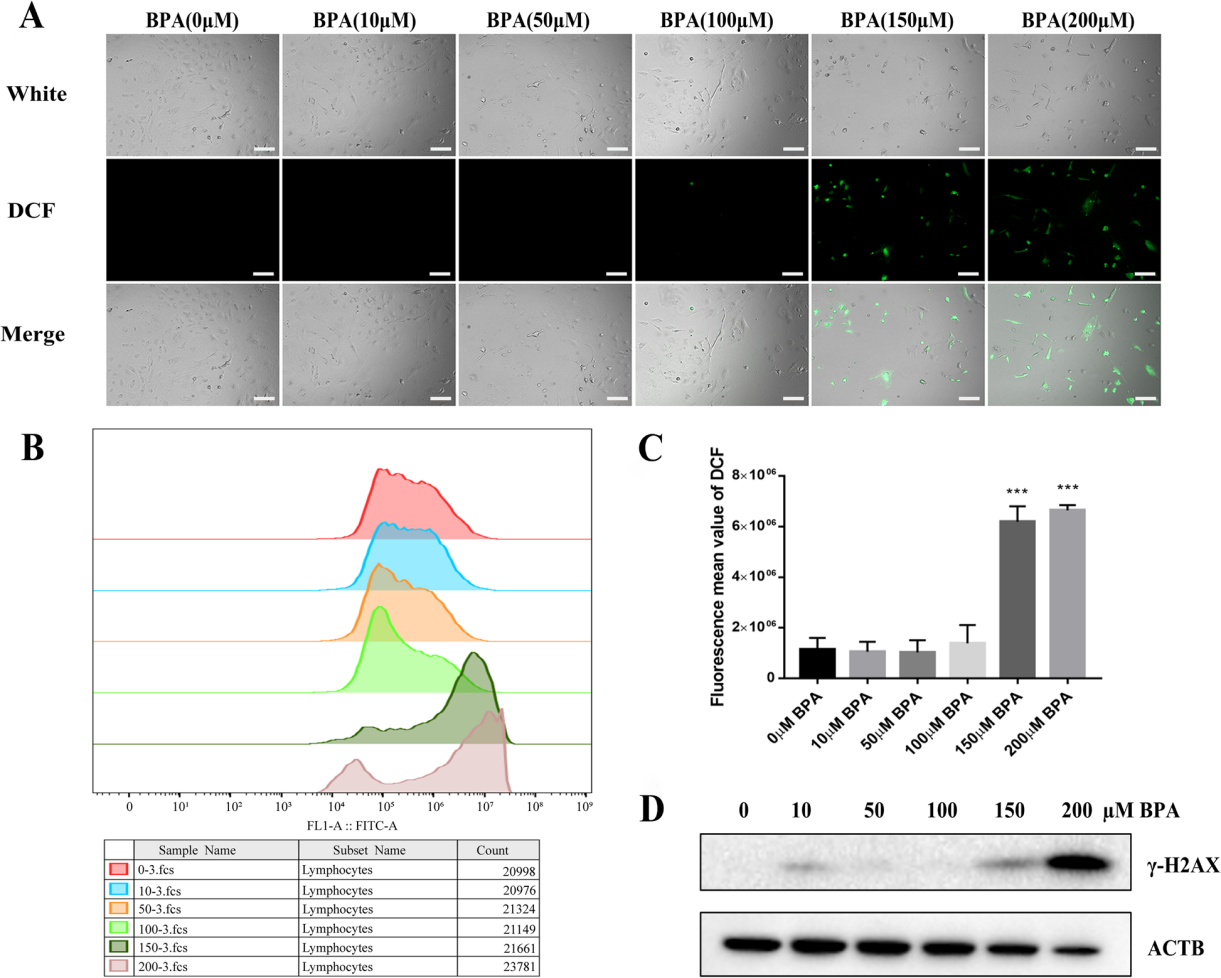
Reactive oxygen species (ROS) levels were detected after high concentrations of BPA (12 h treatment of 200 μM). Fluorescence microscopy (**A**) and flow cytometry (**B**) for the detection of ROS in mpGCs after BPA exposure. **C** shows the results of the statistical figure (**B**). Both fluorescence observation and flow cytometry showed that mpGCs produced a large number of ROS after high concentration and high BPA treatment; (**D**) Western blot results showed that the expression level of γ-H2AX in the high concentration treatment group was obviously higher than that in the control group. ***, *P* < 0.001

**Fig. 4:**
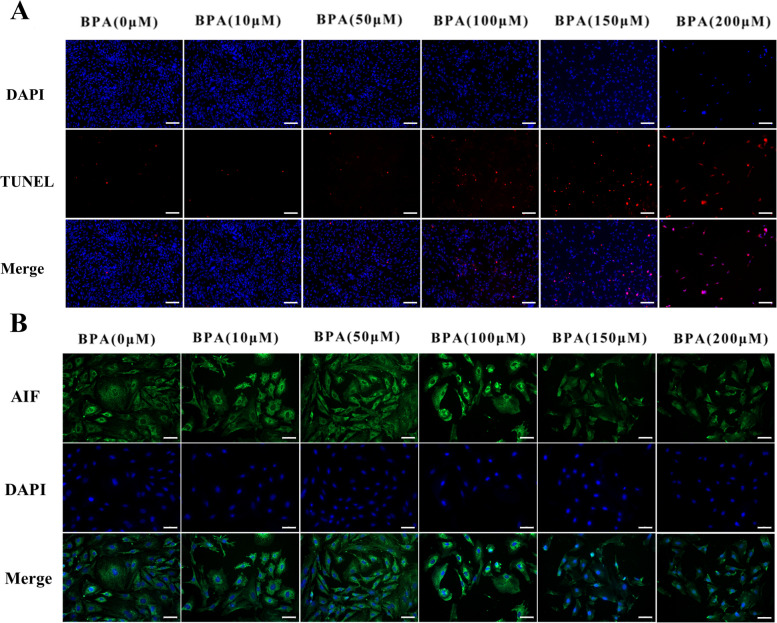
BPA induces apoptosis in mpGCs. mpGCs were treated with gradient concentrations of BPA for 12 h. **A** Terminal deoxynucleotidyl transferase-mediated dUTP-biotin nick end labeling (TUNEL) staining results showed that the high concentration treatment group had a strong apoptotic signal. **B** Immunofluorescence showed a significant translocation of apoptosis-inducing factor (AIF) from the mitochondria to the nucleus in the high concentration treatment group and localization of AIF was detected

Many studies have shown that low doses of reactive oxygen species promote cell proliferation to a certain extent, but exceeding the body's own antioxidant capacity can cause oxidative stress. Reactive oxygen species are mainly composed of mitochondrial respiratory chains, and since the mitochondria of mpGCs were damaged after BPA treatment, a significant amount of intracellular ROS-positive signals was found in the high BPA-treated group (Fig. 3A). The statistical results showed that excessive oxidative stress occurred in the 150 and 200 μM BPA-treated groups (Fig. 3B and C). The results of the TUNEL assay showed that a large number of positive signals were detected in the group that was treated with high concentrations of BPA (Fig. 4A), and the immunofluorescence assay observed that AIF was displaced from the mitochondria to the nucleus with increasing concentrations of BPA (Fig. 4B), which indicated that BPA could induce the production of large amounts of reactive oxygen species in mpGCs and thus trigger apoptosis. The process of apoptosis is accompanied by DNA breakage, and γ-H2AX is a key indicator of DNA damage repair. We examined the expression of γ-H2AX after BPA exposure, and the high concentration of BPA-treated group had a significant positive signal (Fig. 3D).

### Transcriptome analysis of mpGCs after BPA treatment

**Fig. 5:**
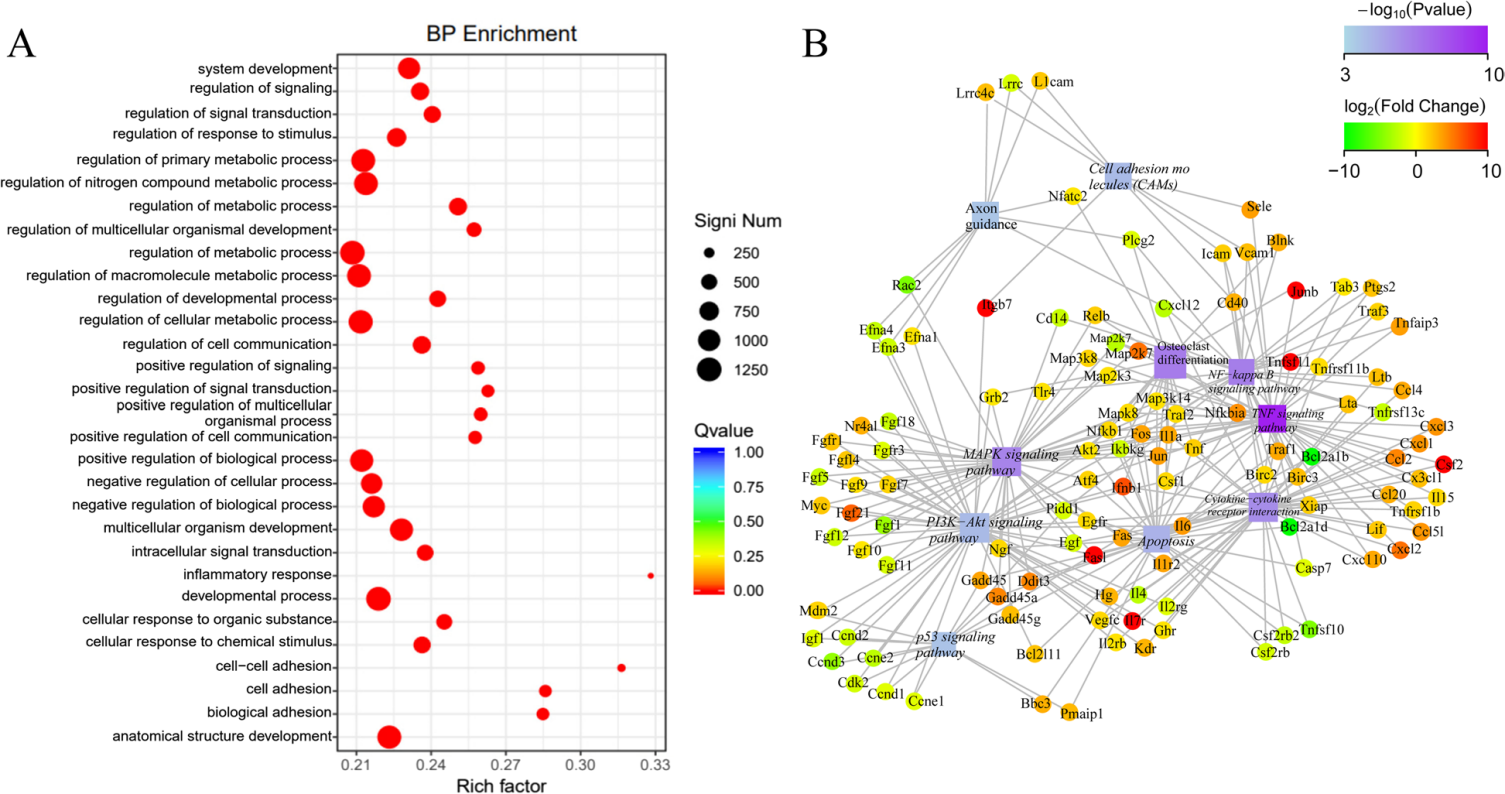
Transcriptome analysis of mpGCs after BPA treatment (12 h treatment of 200 μM). GO enrichment (**A**) and KEGG enrichment (**B**) of differentially expressed genes

To further explore the differences in transcriptome level after BPA treatment of mpGCs, we extracted RNA from the 200 μM BPA treatment group and the control group for high-throughput sequencing. First, the results of RNA extraction were as follows: the results showed that the concentration of RNA extracted was about 800 ng/μl, and the purity met the requirement between 1.8–2.0. GO enrichment analysis (Fig. 5A) showed that the differentially expressed genes between groups obtained by RNA-seq were associated with certain biological processes (system development and regulation of metabolic process), cellular components (nucleosome and DNA packaging complex), and molecular functions (receptor regulator activity). KEGG analysis (Fig. 5B) showed that the differentially expressed genes between groups were involved in p53, MAPK, and PI3K-Akt signaling pathways and the signaling pathways related to apoptosis.

## Discussion

As an EDC that can be absorbed or ingested through daily environmental exposure, BPA can affect human endocrine activity and pose a threat to human health and has been of great interest to researchers for many years [32–34]. Studies showed that BPA is present in higher levels in infertile women than in normal women [14]. BPA may interfere with the embryo implantation process through impacting the morphology and function of the fallopian tubes and uterus [20]. BPA can vary the expression levels of pituitary gonadotropin-related factors and can also interfere with the normal function of the hypothalamic-pituitary-gonadal axis, among other things [35].

BPA in the ovarian follicular fluid, placental tissue, urine, blood, and other tissues can interfere with follicular development and is closely associated with miscarriage, polycystic ovary syndrome, infertility, and other diseases [9, 10, 36]. It is now believed that BPA has weak estrogenic and anti-androgenic activity and can interfere with the normal development of the female ovaries [37]. The female reproductive endocrine system in adolescence is not yet mature. Therefore, the body is susceptible to disturbances by exogenous hormones [38]. In the face of these clinical results, we selected 12 h as the experimental condition to explore the physiological changes caused by BPA at the cellular and molecular levels in a short time, which also provides a new idea for the subsequent drug treatment time. Consequently, it is imperative to study the effect of BPA on early follicular development. BPA is known to have effects through skin contact or entering the body through the respiratory tract. Other studies have set up experimental groups with different concentrations of BPA in the human body, while our study aims to illustrate the effects of exposure to different concentrations of BPA in a short period of time. This has important implications for the future prevention of BPA concentration in the environment. Therefore, the concentration of 200 μM BPA was selected for the simulation treatment and the corresponding experimental results were obtained.

Over a long period of time, a series of studies have been conducted on BPA, from small beetle dapits to mammalian mice to human embryos, and we can find that BPA is far more harmful to living things than we thought [32]. Excessive levels of BPA in daphnia can cause disturbances in the digestive, nervous, and antioxidant systems [39]; Mice exposed to certain doses of BPA have been shown to suffer severe damage in both female and male mice. BPA was more severe during perinatal exposure, causing dysregulation of the hypothalamic-pituitary-ovarian axis in both young and adult mice, leading to axial precocious puberty [40–42]. In addition, exposure to BPA early in life may have intergenerational effects that put offspring at risk for BPA-related diseases [43]. And we know that BPA has been linked to abnormalities in estradiol and impaired ovarian function, such as polycystic ovary syndrome, endometriosis and other diseases. These diseases have caused untold suffering to countless patients and families [44, 45]. With these considerations in mind, we focused our research on mpGCs in follicles, which play an important role in ovarian development, in an attempt to alleviate the clinical symptoms of female infertility through related molecular mechanisms. And so, we looked at oxidative stress inside the cell and tried to combine the two.

Oxidative stress is an important cause of exogenous chemical-induced cellular damage and plays an important role in follicular development disorders [46–48]. In our present study, we successfully observed a significant increase in intracellular ROS signaling after BPA exposure using fluorescence microscopy and flow cytometry. Combined with electron microscopy results, intracellular mitochondrial damage occurred after high concentrations of BPA treatment. Our study suggests that BPA may induce damage to mpGCs by increasing the level of oxidative stress. AIF is a protein with apoptosis-inducing activity that is localized in the membrane gap of mitochondria. When cells are stimulated by apoptosis, AIF molecules are released from the mitochondria and translocated to the nucleus, mediating caspase-independent apoptosis. Our experiments demonstrate that AIF has a significant denuclearization signal after BPA treatment.

In this study, GO and KEGG enrichment analyses were used to predict the functions and potential mechanisms of action of the differentially expressed genes identified by sequencing. The findings showed that these differentially expressed mRNAs have multiple functions, such as promoting reproduction, metabolism, and apoptosis, and may be engaged in various signaling pathways such as p53, MAPK, and PI3K-Akt. Among these signaling pathways, the p53 pathway mediates the regulation of ovarian granulosa cell apoptosis by mRNA molecules, which in turn is involved in the pathogenesis of ovarian-related diseases, such as premature ovarian failure and polycystic ovary syndrome (PCOS) [49, 50]; the PI3K-Akt pathway mediates and regulates biological processes, such as cell proliferation and metabolism and the maintenance of genomic integrity, which in turn affects the recruitment and growth of primordial follicles. The MAPK pathway is also involved in regulating the pathogenesis of PCOS. Therefore, the authors hypothesize that these differentially expressed genes screened by transcriptome sequencing may intervene in the relevant signaling pathways and thus affect the normal developmental process of the ovary.

### Supplementary Information

**Additional file 1.**

**Additional file 2.**

## Data Availability

The data for this study is available from the corresponding author.

## Abbreviations

BPA: Bisphenol A
mpGCs: Mouse pre-antral follicle granulosa cells
ROS: Reactive oxygen species
AIF: Apoptosis-inducing factor
EDC: Endocrine disrupting chemical
TUNEL: Terminal deoxynucleotidyl transferase-mediated dUTP-biotin nick end labeling
DPAI: 4,6-diamino-2-phenyl indole
PI: Propidium iodide
GO: Gene Ontology
KEGG: Kyoto Encyclopedia of Genes and Genomes
PCOS: Polycystic ovary syndrome
