# Supplementary figures and images for: Bisphenol A triggers apoptosis in mouse pre-antral follicle granulosa cells via oxidative stress
Source: J Ovarian Res. 2024 Jan 16;17:20. doi: 10.1186/s13048-023-01322-y (PMC10790560; doi:10.1186/s13048-023-01322-y)

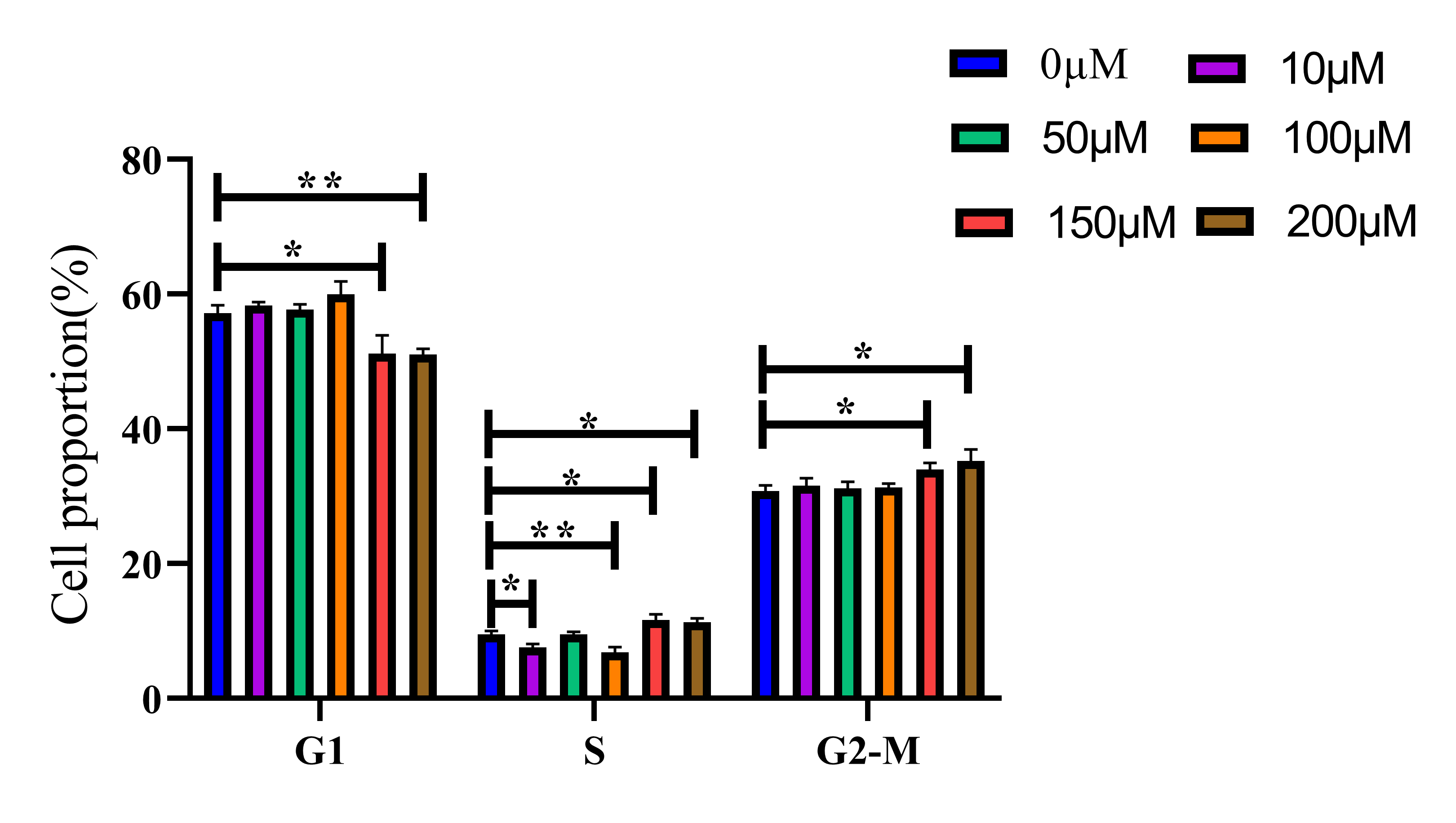

Supplement: Supplementary file 1 — Additional file 1. [file 13048_2023_1322_MOESM1_ESM.tif]
